# INCLUDING PEOPLE WITH COGNITIVE IMPAIRMENT IN HEALTH SERVICES RESEARCH: PROMISING PRACTICES AND LESSONS LEARNED

**DOI:** 10.1093/geroni/igad104.2829

**Published:** 2023-12-21

**Authors:** Alice Prendergast Boughrum, Kristi Fuller

**Affiliations:** Georgia State University, Atlanta, Georgia, United States; Georgia State University, Atlanta, Georgia, United States

## Abstract

Engaging impacted populations, including consumers, in the design of health policies and services is increasingly recognized and prioritized in the United States. However, people with dementia and other forms of cognitive impairment are underrepresented and have often been excluded from health services research. As the number of people worldwide who are living with some form of cognitive impairment is expected to rise dramatically in the coming years, it is critical that researchers across all areas of health services research consider including this population in their work and ensure decisions for exclusion are fully justified. That said, individuals with cognitive impairment constitute a vulnerable population and additional precautions and safeguards are warranted to ensure they are protected throughout and beyond their participation in research studies. The inclusion of people with cognitive impairment also adds complexity to studies, and appropriate research protocols are often challenging to design and implement. This poster will describe two studies conducted by researchers at Georgia State University that included populations with cognitive impairment. The content will cover background about the study populations, which varied in diagnosis and disease progression; methods for recruitment, screening, and consent; and helpful communication strategies to employ throughout the research process. The poster will also share lessons learned and promising practices identified for both studies.

